# Serum Hepcidin Levels, Iron Dyshomeostasis and Cognitive Loss in Alzheimer’s Disease

**DOI:** 10.14336/AD.2016.0811

**Published:** 2017-04-01

**Authors:** Zohara Sternberg, Zihua Hu, Daniel Sternberg, Shayan Waseh, Joseph F. Quinn, Katharine Wild, Kaye Jeffrey, Lin Zhao, Michael Garrick

**Affiliations:** ^1^Department of Neurology, Stroke Center, Buffalo Medical Center, Buffalo, NY, USA.; ^2^Center for Computational Research, New York State Center for Excellence in Bioinformatics and Life Sciences, University at Buffalo, Buffalo, NY, USA.; ^3^Department of Biology, State University of New York at Buffalo, Buffalo, NY 14260 USA.; ^4^Layton Aging & Alzheimer's Research Center, Oregon Health and Science University, Portland, Oregon, USA.; ^5^Department of Biochemistry, State University of New York at Buffalo, Buffalo, NY 14214 USA.; ^6^Department of Pediatrics, State University of New York at Buffalo, Buffalo, NY 14214 USA.

**Keywords:** Alzheimer’s disease, Ferritin, Inflammation, Iron homeostasis, Mild cognitive impairment, Percent transferrin saturation, Serum biomarker

## Abstract

This pilot study examined the status of the master iron regulatory peptide, hepcidin, and peripheral related iron parameters in Alzheimer’s disease (AD) and mild cognitive impairment patients, and evaluated the relationship between iron dyshomeostasis and amyloid-beta (Aβ), cognitive assessment tests, neuroimaging and clinical data. Frozen serum samples from the Oregon Tissue Bank were used to measure serum levels of hepcidin, ferritin, Aβ40, Aβ42 using enzyme-linked immunosorbent assay. Serum transferrin levels were determined indirectly as total iron binding capacity, serum iron was measured and the percent saturation of transferrin calculated. The study variables were correlated with the patients’ existing cognitive assessment tests, neuroimaging, and clinical data. Hepcidin, and iron-related proteins tended to be higher in AD patients than controls, reaching statistical significance for ferritin, whereas Aβ40, Aβ42 serum levels tended to be lower. Patients with pure AD had three times higher serum hepcidin levels than controls; gender differences in hepcidin and iron-related proteins were observed. Patient stratification based on clinical dementia rating-sum of boxes revealed significantly higher levels of iron and iron-related proteins in AD patients in the upper 50% as compared to controls, suggesting that iron dyshomeostasis worsens as cognitive impairment increases. Unlike Aβ peptides, iron and iron-related proteins showed significant association with cognitive assessment tests, neuroimaging, and clinical data. Hepcidin and iron-related proteins comprise a group of serum biomarkers that relate to AD diagnosis and AD disease progression. Future studies should determine whether strategies targeted to diminishing hepcidin synthesis/secretion and improving iron homeostasis could have a beneficial impact on AD progression.

Alzheimer’s disease (AD) is a progressive neurodegenerative disorder with a prevalence of 10% over age 65 and 40% over age 85 [1]. The disease involves progressive loss of memory and language skills and changes in behavior. Among the hallmarks of the disease is extracellular brain deposition of amyloid-beta (Aβ) aggregates which occurs due to either an impaired processing of amyloid-beta precursor protein (AβPP) or an impaired clearance of the Aβ peptide. As a result, Aβ accumulates in the form of plaques. AD pathology also involves the aggregates of hyperphosphorylated microtubular protein, tau, in the form of neurofibrillary tangles.

The e4 allele of apolipoprotein E (APOE4) is a potent risk factor for sporadic AD. Having this specific allele is known to influence AD risk through increased Aβ accumulation [2]. APOE4 can also increase the risk of AD by adversely affecting neuronal integrity through Aβ-independent mechanisms, including neuroinflammation, decrease in neuronal plasticity, and increase in tau phosphorylation [3].

Mild cognitive impairment (MCI) represents an intermediate state where there is overlap between changes attributed to aging, and those fulfilling the criteria for AD dementia. The rate of conversion from MCI to AD is as low as 5-10% or as high as 25-30% [4]. These percentages are influenced by the extent of cognitive impairment at presentation, the presence of an APOE4 allele, the hippocampus and ventricular volumes, the level of brain glucose metabolism as measured by ^18^F-fluorodeoxyglucose positron-emission tomography (PET), and the ratio of Aβ42/tau in cerebrospinal fluid (CSF), detected using the amyloid-binding carbon 11-labeled Pittsburgh compound B tracer and PET [4].

The diagnosis of AD currently depends on a combination of clinical and cognitive assessment tests, but definitive diagnosis of AD requires pathological evaluation at the time of autopsy. Therefore, biomarkers which closely reflect the AD pathology are instrumental for AD diagnosis and for predicting conversion of MCI to AD. Imaging techniques such as magnetic resonance imaging (MRI), functional MRI, and PET have been studied for their suitability in evaluating brain pathology in AD and MCI, but these approaches are not routinely available. In addition, CSF biomarkers are promising given direct contact between CSF and brain’s extracellular space, but due to the invasive nature of lumbar puncture, the use of such biomarkers has limited clinical applications [5].

Serum or plasma biomarkers are relatively simple, less-invasive to obtain; therefore, they have great potential for use in AD. The two Aβ peptides, Aβ_40_, and Aβ_42_ are produced from AβPP cleavage by β-secretase-1 enzyme. Although both forms aggregate, Aβ_42_ is known to be more amyloidogenic than Aβ_40_. These two Aβ peptides are the most studied biomarkers in AD and MCI.

Cross sectional studies report higher plasma levels of either Aβ_42_ or Aβ_40_ levels in AD compared to controls, but there is a broad overlap between the groups, and most studies find no differences among the two groups [6]. Similarly, studies examining the value of plasma Aβ peptides and their ratio in predicting AD in the cognitively normal elderly yield conflicting results [7-9]. These data suggest that AD may require more than a single marker to diagnose the disease accurately or to predict the conversion of MCI to AD, perhaps due to heterogeneity.

Nevertheless, Aβ aggregation and its accumulation in the brain are known to be associated with inflammation and oxidative stress [10]. Under these conditions, radicals can form with the potential for protein and DNA oxidation, as well as lipid peroxidation in AD brain [11]. This inflammation and oxidative stress may also promote brain iron deposition, with the possibility of enhancing Aβ aggregation and toxicity [12]. Increased iron deposition in the cortex and cerebellum is known to occur in the early stage of the disease, in MCI patients [13], and be associated with oxidative stress and early Aβ deposition [14].

There are direct connections between iron homeostasis and AβPP. AβPP has a non-canonical iron response element (IRE) in the 5’ untranslated region of its mRNA [15]. This IRE places translation of the mRNA under the control of iron regulatory proteins (IRPs) such that increased levels of intracellular iron lead to increased amounts of AβPP in a fashion similar to ferritin translation [16]. Of the two IRPs only IRP1 appears to be interacting with this IRE in neurons [17]. Moreover, a sequence within AβPP interacts with ferroportin to improve iron export [18], making AβPP a direct participant in iron homeostasis.

Intracellular iron plays a critical role in many physiological processes. Iron is stored in the cytosolic protein ferritin, whereas iron in the blood in ferric form is bound reversibly to the glycoprotein transferrin [19]. Transferrin has receptors on the BBB endothelial cells, facilitating cellular internalization of iron there [20]. High iron levels in turn stimulate the liver to synthesize the iron regulatory hormone, hepcidin, a 25 amino acid residue peptide, and a key player in iron metabolism. Upregulation of hepcidin leads in turn to decreased release of iron. Conversely when iron stores drop, synthesis of hepcidin is down-regulated, resulting in a cellular release of iron [21].

Hepcidin regulates iron levels by modulating its absorption through enterocytes, its recycling through macrophages, and its storage in hepatocytes [22]. The hormone is also independently produced in regions of the brain [23]. Hepcidin’s regulation of iron is mediated via ferroportin, an iron export protein located on the surface of enterocytes, macrophages and hepatocytes, cells which are capable of releasing iron into plasma for transport by transferrin [24]. Upon release, hepcidin interacts with ferroportin on the cell surface leading to the transporter’s internalization and degradation, preventing cellular export of iron [25]. Inflammation mediated by bacterial lipopolysaccharide and cytokines, especially interleukin-6, upregulate hepcidin synthesis and release; often such a scenario occurs in anemia of chronic disease [26].

Due to the importance of the role of iron dyshomeostasis in AD neuroinflammatory pathology, we chose to determine whether there are differences in the level of serum hepcidin, and related factors (serum ferritin, serum iron, TIBC, % saturation) as potential diagnostic markers in AD and MCI. Although differences in hepcidin levels between AD and control are more likely to be observed in CSF, we hypothesized that effects could be sufficiently systemic that serum levels would be relevant too. In addition, we examined the relationship between iron homeostatic proteins and Aβ_40_ and Aβ_42_ serum levels and their interactions with patients’ available cognitive assessment tests, neuroimaging and clinical data.

## MATERIALS AND METHODS

### Population

The study relied on frozen serum samples obtained from Oregon Brain Tissue Bank. The samples represented 52 AD patients (37 males), age 70.6±7.4 years, 9 MCI (8 males), age 75±11 years, and 24 controls (9 males), age 68.0±9.4 years. Autopsy of 29/52 AD patients confirmed diagnosis of pure AD in 19 patients. The 10 remaining patients had a combination of AD and Lewy body dementia (n=7) or AD and vascular dementia (n=3) (Table 1). All participants in the study (both AD and Controls) were outpatients with an unremarkable general medical exam and complete blood count with no evidence of sepsis, anemia, or any acute illness.

### Materials

Enzyme-linked immunosorbent assay (ELISA) kits for hepcidin (MyBiosource, Cat#, MBS700759, detection range 4.69 ng/ml - 300 ng/ml, and sensitivity of 1.17 ng/ml), Aβ40 (MyBiosource, Cat# MBS727155, detection range 50 pg/ml - 1000 pg/ml, sensitivity: 1pg/ml), Aβ42 (MyBiosource, Cat# MBS729541, detection range 50 pg/ml - 1000 pg/ml, sensitivity: 1pg/ml), ferritin (Abnova, Cat# KA0211, detection range 15 ng/ml - 1000 ng/ml, sensitivity: 5 ng/ml); and the kit for serum iron and unbound iron binding capacity (UIBC) (Stanbio Laboratory, Cat #0370-110) were commercially available.

### Measurements

We measured serum levels of hepcidin, ferritin, Aβ_40_ and Aβ_42_ by ELISA, and calculated the total iron binding capacity (TIBC) as serum iron + UIBC and percent saturation of transferrin assuming TIBC = transferrin. In addition, patients’ cognitive assessment test results, neuroimaging, and clinical data were available for correlation with the studied variables.

### Statistical Analysis

Because we were testing *a priori* hypotheses on hepcidin levels, serum ferritin and iron status rather than engaging in exploratory statistical analyses, we did not correct for multiple tests. Non-parametric tests were used to compared AD and the subgroups, MCI and controls, adjust for differences in autopsy status and post-autopsy diagnosis. Variables and their ratio were calculated and compared between AD patients and controls, pure AD patients and controls, MCI and controls and MCI and AD. *Pearson* correlation was used to test the relationship between study variables and the existing cognitive assessment test results (mini-mental state examination (MMSE), clinical dementia rating (CDR) and CDR-sum of boxes (SOB), neuroimaging (total CNS volume, ventricular CSF volume, hippocampal volume, subarachnoid volume), and clinical (glucose, protein, albumin, white and red blood cells, number of platelets, mean corpuscular volume) data.

### Human Subjects

The study was approved by the Internal Review Board of the State University of New York at Buffalo. The collection of data and samples was approved by the Oregon Health and Science University Internal Review Board.

**Table 1:** Population characteristics

	n	Age (yrs)	MMSE	CDR	CDR-SOB	APOE4 (1 copy)	*P*
Groups							
AD (total)	52 (M=37)	70±7	18.42±6.2	1.085±0.6	6.456±3.0	40 (77%)	
pure AD	19 (M=10)	69±7	16.82±6.3	1.13±0.69	6.46±2.5	14 (74%)	
MCI	9 (M=8)	75±11	29.09±1.5	0.13±0.2	0.1±0.2	2 (22%)	<0.001
Controls	24 (M=9)	68±9	29.25±1.2	0.041±0.1	0.041±0.1	2 (8%)	<0.001

The data are summarized as mean ± SD. Where *P* < 0.001, indicates differences in MMSE, CDR, and CDR-SOB, and the frequency of at least 1 copy of the APOE4 allele between AD patients and MCI, and between AD patients and controls. Abbreviations: AD: Alzheimer’s disease, CDR: clinical dementia rating, MCI: mild cognitive impairment, MMSE: mini-mental state examination, SOB: sum of boxes.

**Figure 1.**
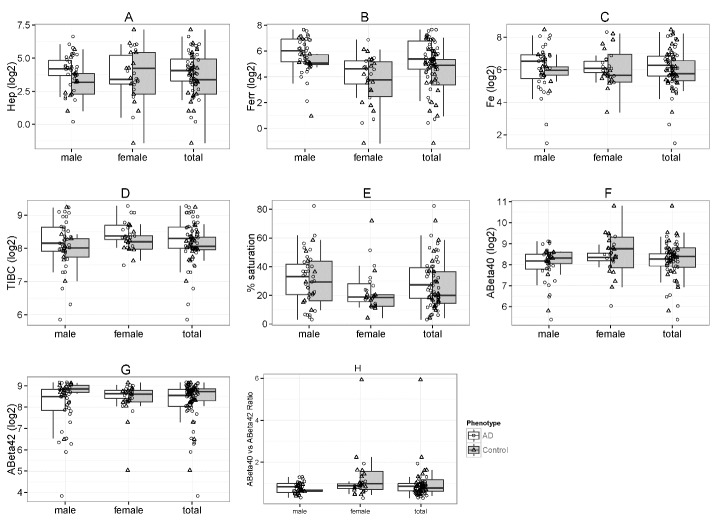
**Distribution of hepcidin, iron-related parameters and Aβ stratified by AD versus controls and by gender (with totals)**. Box and whiskers plots compare serum levels of hepcidin (Hep) (**A**), ferritin (Ferr) (**B**), iron (Fe) (**C**), TIBC (**D**), percent transferrin saturation (**E**), Aβ_40_ (f), Aβ_42_ (g), and Aβ_40/42_ ratio (h).

## RESULTS

Table 1 presents demographics and the severity of the dementia in AD patients, MCI patients, and control subjects, indicated by MMSE, CDR, and CDR-SOB. The ages of the four groups did not differ significantly (all *P* > 0.05). MMSE scores were significantly lower in AD patients (18.42 ± 6.2) as compared to MCI (29.09 ± 1.5) and control subjects (29.25 ± 1.2) (all *P* < 0.001). In addition, about 3/4 of AD patients had a least one copy of the APOE4 allele; while percentages were only 22% and 8% in MCI and control subjects respectively (*P* < 0.001), consistent with the APOE4 allele being a risk factor for AD.

Figure 1 and Table 2 compare serum hepcidin (a), serum ferritin (b), serum iron (c), TIBC (d), percent saturation (e), Aβ_40_ (f), Aβ_42_ (g), and Aβ_40/42_ ratio (h) between AD patients and control subjects, stratified by gender with totals. The figure reveals that the iron-related serum measurements tend to be higher in AD patients compared to controls. Table 2 presents the results as median±SD with associated *P*. While hepcidin, serum iron, TIBC, and % saturation did tend to be higher in AD patients compared to controls, only serum ferritin reached statistical significance with the median slightly more than 1.5x as high (*P* = 0.004). Males had a median more than 2x as high as controls (*P* = 0.05) but the difference in females was not significant for medians, and both AD and control females were lower than males. Furthermore, serum Aβ_40_ and Aβ_42_ levels as well as the Aβ_40_/_42_ ratio exhibited no significant trends.

As a relationship of AD to iron status had emerged, we then chose to see how disease severity interacted. Patients’ stratification based on disease severity, indicated cognitively by CDR-SOB (Table 3) revealed additional connections. The scores for this cognitive assessment test in AD patients ranged from 0-12. We chose to compare AD patients in two strata: CDR-SOB 0-6 and CDR-SOB 6.5-12, to control subjects. Both AD strata exhibited ~1.5x serum ferritin (*P* = 0.003, 0.015), but serum iron levels were ~2x control values (*P* = 0.03), TIBC was ~1.25x control values (*P* = 0.03) for the more demented AD group while the less demented AD patients did not differ significantly from controls. It is worth noting that both hepcidin (*P* = 0.17) and % saturation (*P* = 0.13) were 1.61x higher than controls in AD patients with CDR-SOB ranging from 6.5-12, trends that were not apparent when AD with CR-SOB score of 0-6 compared to control subjects, although neither trend reached significance.

**Figure 2.**
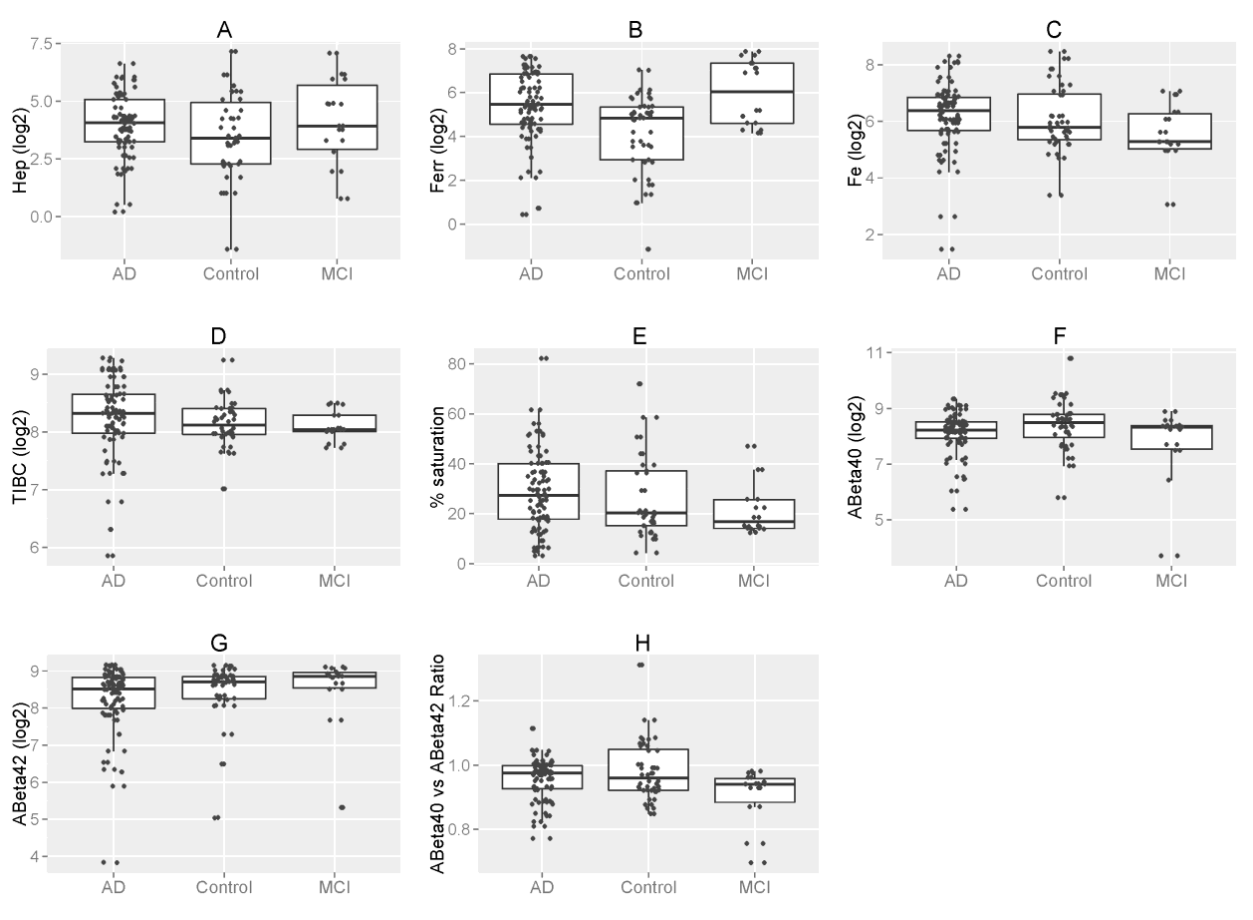
**Distribution of hepcidin, iron-related parameters and Aβ stratified by AD versus MCI and controls**. Box and whiskers plots compare serum levels of hepcidin (Hep) (a), ferritin (Ferr) (b), iron (Fe) (c), TIBC (d), percent transferrin saturation (e), Aβ_40_ (f), Aβ_42_ (g), and Aβ_40/42_ ratio (h).

**Table 2:** Iron and Aβ status for AD *versus* controls, stratified by gender

	Iron and Aβ status for AD *versus* controls, stratified by gender							

Variable	AD (M)	Control (M)	*P*	AD (F)	Control (F)	*P*	AD (T)	*P*
Hepcidin (ngml)	18.31±20.3	9.05±16.4	**0.1**	10.53±24.3	15±38.1	0.9	16.71±21.3	0.28
Ferritin (ng/ml)	68.74±56.1	33.48±35.5	0.1	24.55±28.1	12.79±21.5	0.5	44.19±54.9	**0.004**
Serum Fe (μG/dL)	92.59±69.6	62.5±100.8	0.5	67.7±67.5	51.8±86.5	0.3	83.27±68.4	0.32
TIBC (μG/dL)	304.1±143.4	257.25±135.3	0.6	331.76±119.1	297.35±66.4	0.1	319.78±137	0.25
% Saturation	32.99±18.5	29.25±17.2	0.9	18.81±11.2	19.02±18.2	0.6	27.28±16.8	0.43
Aβ_40_ (pg/ml)	289.08±133.6	317.39±110.9	1	326.34±125.6	428.61±404.6	0.2	297.62±131.5	0.14
Aβ_42_ (pg/ml)	350.03±157.7	461.31±138.7	0.1	394.57±85.7	356.14±132.4	0.3	366.53±141.3	0.21
Aβ_40_/_42_ ratio	0.83±0.27	0.64±0.15	0.2	0.87±0.34	1.01±1.3	0.3	0.86±0.3	0.71

Bold *P* indicate statistical significance.

Abbreviations: Aβ amyloid beta, F female, M: male, T: total, TIBC: total iron binding capacity

**Table 3:** Iron and Aβ status for CDR-SOB (0-6) *versus* CDR-SOB (6.5-12) *versus* controls

Variable	CDR-SOB	CDR-SOB	Ctrls	*P*	*P*	*P*
	(0-6)	(6.5-12)		(0-6 vs.6.5-12)	(0-6 vs. Ctrls)	(6.5-12 vs.Ctrls)
Hepcidin (ngml)	14.34±16.1	17.10±255.7	10.53±31.6	0.396	0.591	0.173
Ferritin (ng/ml)	41.81±61.2	44.49±46.7	28.42±28.7	0.622	**0.003**	**0.015**
Fe (uG/dL)	67.03±58.5	100.55±74.1	55.47±89.9	**0.012**	0.828	**0.032**
TIBC (uG/dL)	300.89±127.1	353.21±139.9	278.21±97.9	0.053	0.926	**0.033**
% Saturation	25.24±15.1	32.87±18.1	20.32±17.7	0.128	0.991	0.134
Aβ_40_ (pg/ml)	289.08±125.2	309.02±131.8	361.44±339.1	0.075	0.064	0.58
Aβ_42_ (pg/ml)	360.81±139.5	372.25±146.4	418.87±137.6	0.836	0.412	0.288
Aβ_40_/_42_ Ratio	0.83±0.2	0.87±0.3	0.80±1.1	0.118	0.267	0.548

Bold *P* indicate statistical significance.

Abbreviations: As in Table 2 and Ctrl: controls

MCI frequently converts to full blown AD although the frequency is not so high that one has strong *a priori* expectations of finding early AD indicators, Nevertheless, Figure 2 and Table 4 compare the study variables for MCI patients *versus* controls and for MCI patients *versus* AD patients to learn if an early sign is present. Serum ferritin was 2.7x as high in MCI as in controls (*P* = 0.025), suggesting that it could be an early indicator of the potential for increasing dementia. No other variable exhibited a significant difference for MCI-control comparisons.

**Figure 3.**
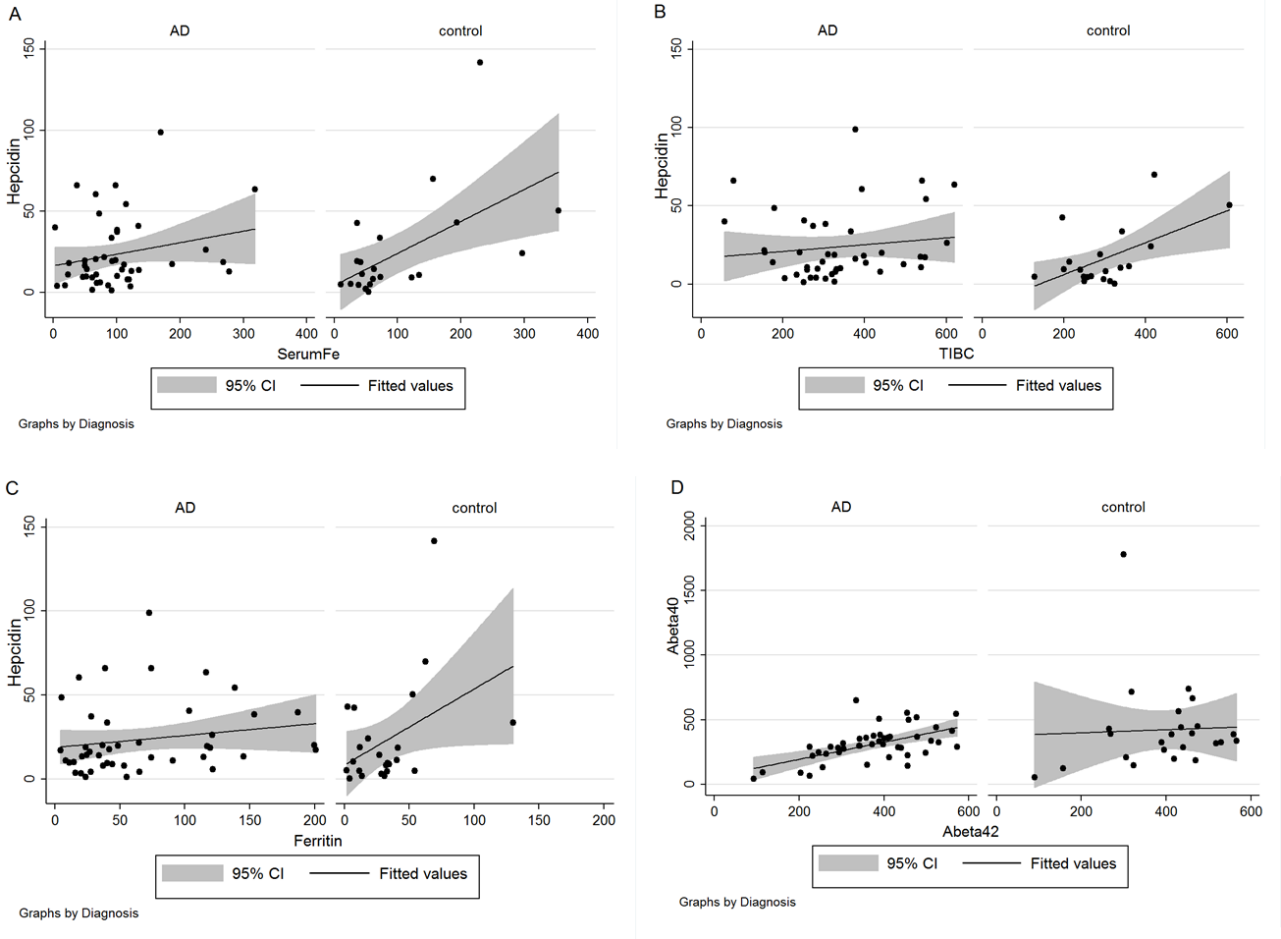
**Correlations between measurements depend on diagnosis**. For each panel, lines of regression indicate the existence or absence of a correlation and the shaded region shows the 95% confidence interval (CI) with data stratified by diagnosis of AD or control for hepcidin *versus* iron (A), hepcidin *versus* TIBC (B), hepcidin *versus* ferritin (C), and Aβ_40_ *versus* Aβ_42_ (D).

**Figure 4.**
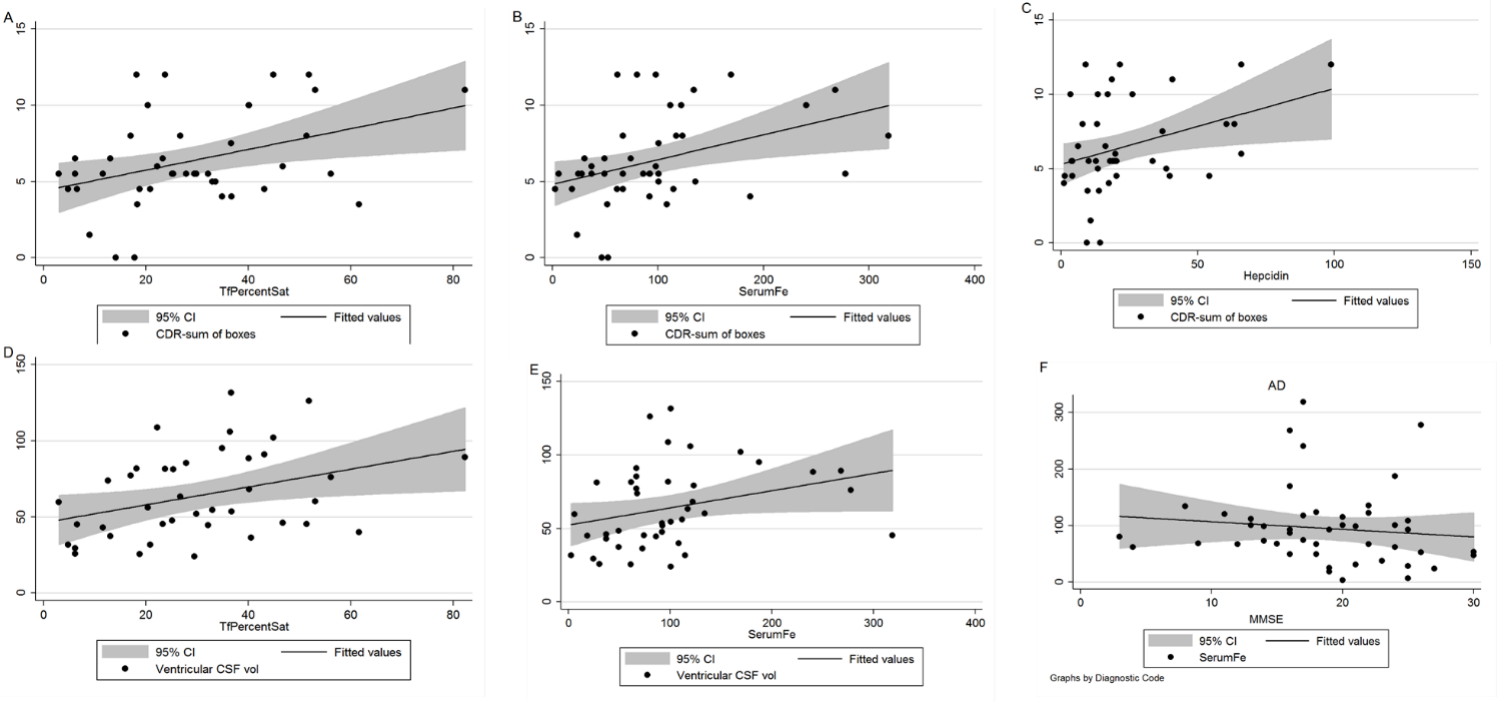
**Disease severity correlates with iron-related measurements in AD**. Scatter plots with regression lines and shaded regions for the 95% confidence interval (CI) for % transferrin saturation *versus* CDRSOB (A), serum iron *versus* CDRSOB (B), hepcidin *versus* CDRSOB (C), % transferrin saturation *versus* ventricular CSF volume (D), serum iron *versus* ventricular CSF volume (E) and serum iron *versus* MMSE (F).

Comparison between MCI and AD patients showed 1.75 x higher serum ferritin in the former also suggesting that it could be an early indicator of increasing dementia but this difference did not reach significance (*P* = 0.36). It could be worthwhile to follow MCI patients longitudinally to see if there is predictive value in serum ferritin data. Furthermore, there was a significantly lower Aβ_40_/_42_ ratio (0.69 ± 0.2 vs. 0.86 ± 0.3, *P* = 0.026) in MCI patients compared to AD patients, probably reflecting higher values for the denominator that did not themselves reach significance (462.60 ± 154.5 pg/ml vs. 366.53 ± 141.3 pg/ml, *P* = 0.09). No other variable exhibited a significant difference for MCI-AD comparisons although serum iron was ~47% higher in the AD as compared to MCI (*P* = 0.08), suggesting that this measurement could also be of interest as rising only in genuine AD if one follows MCI patients longitudinally.

**Table 4:** Iron and Aβ status for MCI *versus* controls and MCI *versus* AD.

Variable	MCI	Control	AD (T)	*P* (MCI-Control)	*P* (MCI-AD)
Hepcidin (ngml)	15.1±40.5	10.53±31.6	16.71±21.3	0.397	0.769
Ferritin (ng/ml)	77.52±83.5	28.42±28.7	44.19±54.9	**0.025**	0.363
Fe (uG/dL)	38.86±39.8	55.47±89.9	83.27±68.4	0.241	0.083
TIBC (uG/dL)	263.13±50.7	278.21±97.9	319.78±137	0.93	0.161
% Saturation	16.75±11.6	20.32±17.7	27.28±16.8	0.64	0.227
Aβ_40_ (pg/ml)	321.41±134.4	361.44±339.1	297.62±131.5	0.14	0.373
Aβ_42_ (pg/ml)	462.60±154.5	418.87±137.6	366.53±141.3	0.33	0.091
Aβ_40_/_42_ Ratio	0.69±0.2	0.80±1.1	0.86±0.3	0.122	**0.026**

Bold *P* indicate statistical significance.

Abbreviations: As in Table 2

**Table 5:** Correlations to cognitive assessment tests, neuroimaging, and clinical data in AD patients.

Variables	*r*	*P*
*Cognitive assessment tests*		

Serum Fe-CDRSOB	0.387	**0.009**
Serum Fe-CDR	0.361	**0.015**
Serum Fe-MMSE	-0.269	0.064
%Saturation-CDR	0.355	**0.019**
%Saturation-CDRSOB	0.319	**0.042**
Hepcidin-CDRSOB	0.329	**0.03**
Hepcidin-MMSE	-0.253	0.09
Aβ40-CDRSOB	0.321	**0.04**

*Neuroimaging*		

Serum Fe-Vent. CSF Vol	0.4	**0.009**
%Saturation-Vent. CSF Vol	0.383	**0.015**

*Clinical*		

Hepcidin-Glucose	0.408	**0.007**
Hepcidin-Protein	0.373	**0.01**
%Saturation-MCV	0.295	0.051
APOE-Aβ40/42 ratio	-0.327	**0.032**

An unexpected loss of correlations appeared in AD when we compared AD patients and controls (Fig. 3) for associations between hepcidin and other iron-related parameters. The correlations between hepcidin, and iron (*r* = 0.228, *P* = 0.13) (Fig. 3A), hepcidin and TIBC (*r* = 0.133, *P* = 0.39) (Fig. 3B), and hepcidin and ferritin (*r* = 0.18, *P* = 0.23) (Fig. 3C) were not statistically significant in AD patients, whereas in control subjects, those between hepcidin and iron (*r* = 0.57, *P* = 0.01), hepcidin and TIBC (*r* = 0.56, *P* = 0.01), and hepcidin and ferritin (*r* = 0.41, *P* = 0.05) all remained significant. The breakdown of the significant relationship between iron-related markers may be a part of AD pathology. In contrast, AD patients showed significant correlation between Aβ_40_ and Aβ_42_ (*r* = 0.57, *P* <0.001) (Fig. 3D), whereas this association was absent in controls (*r* = 0.0423, *P* = 0.84).

Table 5 presents the relationship between study variables and patients’ cognitive assessment tests, neuroimaging, and clinical data, by noting the calculated *r* and *P* values for those correlations that reached statistical significance or near-significance. We observed a significant correlation between iron and CDR-SOB (*r* = 0.387, *P* = 0.009), iron and CDR (*r* = 0.361, *P* = 0.015), between hepcidin and CDR-SOB (*r* = 0.329, *P* = 0.03), between %Saturation and CDR (*r* = 0.355, *P* = 0.019), and %Saturation and CDR-SOB (*r* = 0.319, *P* = 0.04); In addition, Aβ_40_ correlated with CDR-SOB (*r* = 0.321, *P* = 0.04). The relationship between iron and MMSE (*r* = - 0.269, *P* = 0.06), and hepcidin and MMSE (*r* = - 0.253, *P* = 0.09) had similar trends that only reached near-significance. The correlation between iron-related proteins and neuroimaging data demonstrated a significant association between serum iron and ventricular CSF volume (*r* = 0.420, *P* = 0.009), and between % transferrin saturation and ventricular CSF volume (*r* = 0.383, *P* = 0.015).

In addition, hepcidin correlated with plasma glucose (*r* = 0.408, *P* = 0.007) and protein (*r* = 0.373, *P* = 0.01) levels. We observed an inverse association between Aβ40/42 ratio and the presence of higher APOE4 allele (*r* = - 0.327, *P* = 0.032). Similar analysis in control subjects did not show a significant association between iron-related proteins and cognitive assessment tests, neuroimaging, and clinical data (results not shown).

We have also chosen to depict some of the correlations between disease severity and iron-related measurements in Figure 4. These correlations are graphed for % saturation and CDRSOB (A), serum iron and CDRSOB (B), hepcidin and CDRSOB (C), % saturation and ventricular CSF volume (D), serum iron and ventricular CSF volume (E), and serum iron and MMSE (F).

## DISCUSSION

### Serum ferritin

This study tested the hypothesis that iron dyshomeostasis might be apparent even via peripherally accessible measurements of hepcidin and parameters related to iron metabolism in AD patients compared to MCI patients, who might also exhibit a usually lesser level of dyshomeostasis, compared to control subjects. In addition, we compared the value of iron related serum markers, as diagnostic tools to those of Aβ peptides. We found that group differences in serum ferritin were statistically significant. The stratifications in particular suggest that this marker, although not specific for AD and challenging to separate whether its attribution is to inflammatory problems or iron dyshomeostatsis, is one that appears early in AD evolution. Thus it could be useful in early diagnosis of AD in combination with other markers

Ferritin, itself, is primarily an intracellular iron storage protein, and serum ferritin usually reflects liver iron stores. Serum ferritin, however, is an acute phase indicator so also a marker of inflammation as the byproduct of leakage of apo-ferritin into the serum from damaged cells. Iron released and left behind then has potential for causing more cellular toxicity and damage [27]. Our ELISA measurement of ferritin does not distinguish between iron-liganded and apo ferritin, but there is a little used assay that could do this [28]. Usually serum contains significant levels of iron-liganded ferritin, in addition to the unliganded form [29, 30]. Ordinarily, the major cellular source of serum ferritin is hepatic. Macrophages play a critical role in iron homeostasis, by recycling iron from red blood cells and storing it in ferritin prior to reutilization by developing erythroid cells. Ferritin is also expressed in lymph node macrophages as well as in alveolar macrophages of the lung, and sinus macrophages of the liver and spleen [31]. Murine studies using mass spectrometry and immunoblotting techniques show a regulated lysosomal ferritin secretion by macrophages into the serum; this active secretion can be a major source of iron-liganded serum ferritin [30], with physiological role as iron donor to developing erythroid cells [31, 32]. In AD patients serum ferritin levels may also be attributed to leakage from the brain due to compromise in the BBB integrity [33].

Although brain iron overload (perhaps only regionally) may explain part of the increase in AD ferritin serum levels, higher serum ferritin levels are also observed in MCI patients, and in AD patients at early stages of the disease, stages where there are relatively low serum iron levels. These results suggest inflammation as the likely cause of the observed increase in serum ferritin in AD patients [34]. Indeed, both CNS and systemic inflammation have been documented in early stages of AD, contributing to the progression and the pathogenesis of the disease [35, 36]. It is also noteworthy that unlike the increase in ferritin serum levels observed at dormant and early stages of the disease, the increase in serum iron levels seems to characterize a later stage in the AD pathology, suggesting that these two events may occur independent of each other.

Ferritin consists of two main subunits, H and L subunits, for heavy and light, reflecting their molecular weight. Among the transcriptional mechanisms controlling the H subunit is an upstream enhancer responsive to NF-κB [37, 38] and other signal systems [39, 40] so that H subunits increase during inflammation and oxidative stress. Both the H and L-subunit also respond translationally to high iron levels and oxidative stress [41]. These characteristics account for the involvement of ferritin in modulating inflammation and production of reactive oxygen radicals.

Cell type and physiologic function are among factors determining the H to L ratio. Changing conditions such as cell differentiation, inflammation, infection, and other environmental stress can modulate this ratio. The differences in H to L ratios can give rise to diverse isoferritins, each possessing distinct metabolic properties [42, 43]. A higher H to L ratio is known to be synthesized in cases of inflammation and oxidative stress to curb these pathological processes [40]. This response is apparent in frontal cortex of AD patients, who demonstrate three times higher H/L ratio than age-matched controls [44]. However, this ratio is lower in globus pallidus of AD patients compared to controls [44], suggesting region specific brain disturbances in H/L ratio. AD patients also do not demonstrate age-induced increases in H and L protein subunits, in many parts of the brain, including caudate, putamen and substantia nigra, as the control subjects do [44].

A dysregulation in the H/L ratio would have the potential contributing to neuroinflammation and oxidative stress, and lead to suboptimal H-subunit derived ferroxidase activity, and higher levels of unliganded iron with the potential for cellular damage. Macrophages are known to increase their ferritin synthesis, predominantly that of the H subunit, upon activation [40]. Peripheral macrophages are known to be activated in AD patients [45]; likely reflecting similar microglial activation. H to L ratio dysregulation in ferritin and the inability to curb inflammation and oxidative stress may contribute to higher systemic inflammation observed in early stages of AD [36].

### Peripheral evidence for iron dyshomeostasis and AD pathology

While the etiological distance between peripheral indicators of iron metabolism and actual disturbed iron metabolism in the brain may be so great that confounding effects prevented us from detecting significant relationships when comparing AD patients to controls, we observed such relationships between serum iron and cognitive assessments of AD severity, including CDR and CDR-SOB. These are considered to be more precise measures of cognitive dysfunction than MMSE [46]. Similarly, hepcidin and % transferrin saturation correlated with these cognitive assessment tests. These results suggest that levels of iron and other iron-related serum proteins may reflect the levels in the brain [47]. Consistent with this assumption, brain MRI evaluation of iron levels, measured by proton transverse relaxation rate, has been shown to correlate with both iron serum levels and % transferrin saturation [47]. It is of particular interest that some correlations that occur in aging controls break down in AD (Figure 3). This observation combined with the others in our results suggests that there are at least two kinds of pathological events in AD - those associated with iron dyshomeostasis and those associated with a process that disrupts normal associations of iron-related parameters during aging. This disruption is also apparent in the light of significant increase in serum ferritin, without significant accompanied increases in serum hepcidin and / or iron. The reason for this disruption is unknown but may be related to AD inflammatory signaling.

We observed correlations between ventricular CSF volume and serum iron and between ventricular CSF volume and % transferrin saturation (Table 3). Ventricular CSF volume is an early MRI marker of cognitive decline and a quantitative method to evaluate neuropathological changes associated with AD [48-50]. Clearly our study needs to be repeated and extended in such a fashion to learn enough about iron dyshomeostasis and inflammatory indicators in AD to decide on interventions that could lead to an improved outlook for AD patients. Additional supporting data on iron dyshomeostasis in AD would be required to determine whether interventions targeted to promote iron homeostasis, such as dietary iron restriction/iron chelation [51] could lead to improved clinical outcomes in AD patients. Strategies targeted to diminishing hepcidin synthesis/secretion could also lead to improved clinical outcomes in AD patients and warrant further investigations. Nevertheless, our observation of the loss of correlation between serum hepcidin and iron levels in AD patients suggest that iron dyshomeostasis in AD patients may extend beyond alterations in serum levels into a disruption of hepcidin regulatory control. Similarly, a loss of association has been also reported between ferritin H and L subunits in AD [44], indicating the complexity of the AD pathology. Therefore, AD treatment modalities may exert independent clinical effects and possibly synergize when combined.

### Aβ40 and Aβ42 in AD patients

The observation of a strong association between Aβ40 and Aβ42 has been also reported in an earlier AD study [52]. This association may partially explain the involvement of both AβPP products in the pathology of AD and their aggregation and co-localization in the plaques. Nevertheless, no significant correlation between iron-related proteins and Aβ’s was identified. This observation agrees with the results of an MRI study of sample slices from the entorhinal cortex of AD patients showing that the transverse relaxation rate associated with amyloid plaques is independent from that of iron load [53]; this in turn also suggests that treatment modalities to correct iron dyshomeostasis may be independent and synergize with those targeted in reducing AD plaques.

Earlier studies show that Aβ_40 and_ Aβ_42_ plasma levels do not correlate with CSF levels of these proteins in AD patients [54]; moreover, no significant correlation between Aβ_40_ and Aβ_42_ plasma levels and cognitive assessment tests and neuroimaging data has been demonstrated [52]. These studies and our results showing a weak association between Aβ serum levels and CDR-SOB severity suggest that despite long standing interest, Aβ peptides may be suboptimal biomarkers in AD routine clinical practice in general, and in estimating AD disease progression in particular. Nevertheless, the significantly low Aβ_40/_Aβ_42_ ratio in MCI patients, which stems from an early rise in Aβ_42_, preceding its decline, may have potential diagnostic power.

### Hepcidin and glucose

We also found a correlation between serum hepcidin and plasma glucose levels. An early name for hemochromatosis was the bronze diabetes, reflecting damage to the pancreas by iron overload so there are earlier reports that are relevant such as a higher incidence of diabetes in relation to body iron stores [55, 56]. Peripheral insulin resistance is a common finding in patients with hemochromatosis [57]. Extrahepatic sources of hepcidin include pancreatic β-cells, where insulin and hepcidin appear to colocalize [58]. As a result, the glucose induced insulin secretion from pancreatic cells, can also lead to hepcidin secretion into the plasm and serum iron modulation [59]. The higher than normal serum hepcidin may explain the hypoglycemia observed in AD patients [60] and the association between hypoglycemia and risk of AD [61].

### A discrepant study

While we were analyzing the data, a paper appeared that finds decreased plasma iron and lower transferrin saturation in AD patients than in controls [62]. The methods were very different and the patients were Australian, but it is difficult to reconcile their results with ours. Our study limitations should be taken into consideration too. First, the number of patients’ samples, especially those with MCI or with pure AD were relatively small. Our results should be repeated in larger cohort and both our methods and those applied to the Australian series should be compared probably without confining patients and controls to a specific geographic area of Oregon. It would also be of interest to have non-Caucasian patients.
